# Newcastle Disease Virus Infection Interferes With the Formation of Intestinal Microflora in Newly Hatched Specific-Pathogen-Free Chicks

**DOI:** 10.3389/fmicb.2018.00900

**Published:** 2018-05-07

**Authors:** Ning Cui, Xiaoying Huang, Zhengjie Kong, Yanyan Huang, Qinghua Huang, Shaohua Yang, Lin Zhang, Chuantian Xu, Xiumei Zhang, Yanshun Cui

**Affiliations:** ^1^Shandong Key Laboratory of Animal Disease Control and Breeding, Institute of Animal Science and Veterinary Medicine, Shandong Academy of Agricultural Sciences, Jinan, China; ^2^Shandong Provincial Key Laboratory of Animal Biotechnology and Disease Control and Prevention, Shandong Agricultural University, Tai’an, China

**Keywords:** Newcastle disease virus, 16S rRNA sequencing, duodenal microflora, cecal microflora, dysbiosis

## Abstract

Newcastle disease virus (NDV) infection leads to disproportion of intestinal tract microbiol population in chickens. Whether vertical infection of NDV affects the formation of a healthy and diverse intestinal community in newly hatched chicks, which might further perturb the establishment of a normal intestinal mucosal immunity, is unclear. This study examined the effects of NDV infection of chick embryos on the formation of the intestinal microbiome of chicks at hatch using 16S rRNA genes pyrosequencing. Eleven-day-old specific-pathogen-free chicken eggs were inoculated via intra-allantoic way with Class I NDV strain. At hatch, chicks were randomly selected and their duodenal and cecal contents were extracted and examined for the composition of gut microflora by Illumina sequencing of the V3+V4 region of the 16S rRNA genes. The results showed that the duodenal flora possesses a greater sample richness and higher microbial diversity as compared with the ceca flora in newly hatched chicks. In addition, there is a clear association with loss of important bacterial population in concert with an enrichment of potentially pathogenic population and NDV infections, both in the duodenum and ceca. It is also increasingly observed that the NDV infection may be associated with the dysbiosis of gut flora. This study presented a profile of the early intestinal microbiota in specific-pathogen-free chicks at hatch and strongly indicates that NDV infection interferes with the formation of intestinal microbiome in newly hatched chicks.

## Introduction

The microflora in the gastrointestinal tract (GIT) of chickens play an important role in nutrition, detoxification of certain compounds, growth performance, and protection against pathogenic bacteria (Kelly et al., 2005; Roto et al., 2016; Varmuzova et al., 2016). Successions occurred from a transient community to one of increasing complexity as the birds aged (Lu et al., 2003). In ovo inoculation of chicken embryos with probiotic bacteria led to the establishment of a healthy and diverse community of microorganisms to colonize the developing GIT (de Oliveira et al., 2014; Roto et al., 2016). However, insights into the interaction occurring between host and bacteria, as well as the influence of environmental factors especially pathogens on the formation of microbial communities in the GIT of chickens are still lacking.

The intestinal microflora is relatively stable under common circumstances, but is easily influenced by various diseases (Barnes, 1979; Gong et al., 2007; Ma et al., 2017). Since bacteria may play a role in the disease process due to the modification of intestinal innate immunity by dysbiosis and translocation of bacteria, changes of normal microflora in the digestive tract are important sites in the susceptibility of chickens to bacterial infection (Mead and Adams, 1975; Barnes, 1979; Stanley et al., 2014). Simian immunodeficiency virus (SIV) infection of chimpanzees led to the disorder of gut communities marked by temporally stable microflora structure and followed by resultant microbial translocation with accelerated rate of change in gut microbiota composition (Moeller et al., 2013). Newcastle disease virus (NDV) is the causative agent of Newcastle disease (ND), which is one of the most highly contagious diseases in chickens, and result in severe economic losses to the poultry industry worldwide (Alexander, 1989; Turmagambetova et al., 2017). It has been reported that NDV infection of chicks induced disproportion of GIT microbiol population and the influence of NDV became more serious with time going on by ERIC-PCR based fingerprints of total DNA from fecal samples (Li, 2007).

Polymerase chain reaction (PCR)-based culture-independent methods have been employed and the diversity and complexity of the intestinal microbiota were much higher than had been reported previously by culture-based studies (Handelsman, 2004). To better characterize the interaction between NDV and host from the perspective of intestinal flora, we performed Illumina high-throughput sequencing of the V3+V4 region of the 16S rRNA genes to examine and analyze the composition of duodenal and cecal microflora in the newly hatched chicks under NDV infection of chick embryo. The profile of early intestinal microbiota in duodenum and ceca of chicks at hatch, and the dysbiosis of bacteria associated with NDV infection were shown in our study.

## Materials and Methods

### Virus and Cultivation

The plaque-purified NDV isolate (Class I) SD21/13 was originally isolated in 2013 and propagated using SPF chicken eggs (Huang et al., 2015). The mean death time (MDT) value of strain SD21/13 in embryonated chicken eggs was >120 h, and the intracerebral pathogenicity index (ICPI) in 1-day-old chickens was 0.18. The virus is effectively proliferated in the chick intestine and induced shed of the epithelial cells from the duodenal intestinal mucosa with lymphocyte infiltration in the intrinsic layer. The purified viruses were tested negative by RT-PCR and excluded the existence of avian influenza virus (AIV), infectious bronchitis virus (IBV), and infectious bursal disease virus (IBDV). The virus-containing allantoic fluids harvested was titrated as described elsewhere (King, 1999) and stored at -80°C until use.

### Experiment Design

SPF chickens were used to avoid the infection of other vertical pathogens. Prepared NDV culture was inoculated into 20 11-day-old SPF chicken eggs via intra-allantoic way with 1 × 10^6^ 50%-egg-infectious doses (EID_50_) per egg. Ten other SPF chicken eggs were inoculated with sterile PBS and used as un-infected controls. All eggs were incubated at 37.5°C (standard temperature) with 65% humidity. Eggs were candled daily and early death embryos in the first 24 h were removed. Once the eggs have hatched, three birds were taken from the NDV infected and un-infected group. Day-old birds were killed by cervical displacement, and in a laminar flow cabinet, the GIT was exposed. Using sterile surgical knife and forceps, the duodenum and ceca of chicks were collected into microtubes and properly stored at -80°C.

### Extraction of Total DNA

DNA was extracted from intestinal samples including the contents and the mucosal wall of the duodenum and ceca from the each chicken according to the protocol described by the E.Z.N.A Soil DNA Kit (OMEGA). Acquired DNAs were measured for their quantities and integrity by Nanodrop ND-1000 spectrophotometer (Thermo Scientific) and 1% agarose gel electrophoresis, respectively. The range of DNA size was measured using the standard low DNA mass ladder and then was stored at -20°C until further processing.

### 16S rRNA Amplification and MiSeq Sequencing

A gene region about 415 nt, covering the V3–V4 region of the 16S rRNA gene was selected to construct community library through tag pyrosequencing. The target gene was firstly amplified using broadly-conserved primers Amplicon PCR Primer F (5′-*TCGTCGGCAGCGTCAGATGTGTATAAGAGACAG*TACGGRAGGCAGCAG-3′) and Amplicon PCR Primer R (5′-*GTCTCGTGGGCTCGGAGATGTGTATAAGAGACAG*AGGGTATCTAATCCT-3′). The primer pairs contain transposase sequences as indicated above in italicized letters. PCR were carried out in a 25 μl reaction containing 2× KAPA HiFi HotStart ReadyMix, Amplicon PCR Primer F (1 μM), Amplicon PCR Primer R (1 μM), and 5 ng DNA template. The PCR condition were initial denaturation at 95°C for 3 min, followed by 25 cycles of denaturation at 95°C for 30 s, annealing at 60°C for 30 s and extension at 72°C for 30 s, with a final extension phase at 72°C for 10 min. PCR products were purified with 0.8 times of AMPure XP Beads. The second round of the PCR was carried out using the index primer pairs Index 1 Primer (5′-AATGATACGGCGACCACCGAGATCTACAC[i5]TCGTCGGCAGCGTC-3′) and Index 2 Primer (5′-CAAGCAGAAGACGGCATACGAGAT[i7]GTCTCGTGGGCTCGG-3′) containing P5/P7 adaptors and 8 nt of index sequences. PCR were then carried out in a 50 μl reaction. Illumina MiSeq was performed on a PE300 instrument commercially at Shanghai Hanyu Bio-Tech, on a MiSeq Sequencer platform.

### Bioinformatic Analysis of Sequencing Data

PE reads get from MiSeq sequencing were spliced according to overlap relationships. Quality of the sequence was evaluated with filtration and quality control to obtain high-quality sequences. All of the sequences from all of the samples were clustered into operational taxonomic units (OTUs) based on a 97% identity threshold. The most abundant sequence of each OTU (97% similarity) was BLAST searched against the Greengene/sliva databases to determine the phylogeny of the OTU. A variety of diversity index analyses were carried out on the basis of OUT and OUT clustering results. Metastats analyses of community structure at the genus level were carried out on the basis of taxonomist analysis using mothur v.1.32.1 (Schloss et al., 2009).

### Statistical Analyses

Differences between populations had been analyzed using parametric (ANOVA) and non-parametric statistical methods. All results were presented as the mean value (± SE). Differences between groups were declared significant at *p* < 0.05.

## Results

### Description of the Sequencing Data

Samples were obtained from the duodenum and ceca of chickens in control group and chickens infected with NDV. After two separate runs on a PE300 instrument and quality-filtering as described in the methods, 123, 730 total sequences with an average of 415 bp in length were available and used for analysis. The sequences were rarified to the minimum number of high quality sequences in all samples and normalized by total count for the alpha and beta diversity analyses as conducted below.

### Establishment of Duodenal and Cecal Flora in the Newly Hatched Chicks

The sample richness was assessed by OTU counts in each individual sample as shown in **Table 1**. The number of observed OTUs in the duodenum and ceca samples were 540 and 273, respectively, in the newly hatched chicks. Consistent with that, duodenal flora also showed a higher sample richness as reflected by the abundance index Ace and Chao and greater bacterial diversity as assessed by both the Shannon and Simpson analyses.

**Table 1:** Number of OTUs per groups and estimators of diversity and richness.

Sample	Reads	OTUs	ACE	Chao	Shannon	Simpson	Coverage
C.D.1	10223	808	3470.821	2464.014	3.365	0.222225	0.953
C.D.2	10040	416	1237.866	930.077	3.792	0.132827	0.984
C.D.3	10061	395	761.817	806.552	2.561	0.366697	0.985
N.D.1	10884	328	521.129	527.588	2.466	0.228863	0.989
N.D.2	10767	434	891.395	1349.056	2.892	0.204073	0.983
N.D.3	9797	727	2348.15	1866.069	3.915	0.123335	0.963
C.C.1	10554	96	807	579	0.751	0.65377	0.993
C.C.2	10020	131	540.313	311	0.662	0.791132	0.992
C.C.3	11454	592	2918.133	1649.467	4.796	0.028698	0.973
N.C.1	9774	572	1162.361	1031.058	4.180	0.102245	0.978
N.C.2	9464	153	1152.913	524	1.368	0.448812	0.989
N.C.3	10692	174	817.224	439.435	1.490	0.375525	0.990


*C.D., duodenum of control chicks; N.D., duodenum of NDV infected chicks; C.C.,ceca of control chicks; N.C., ceca of NDV infected chicks.*

**FIGURE 1:**
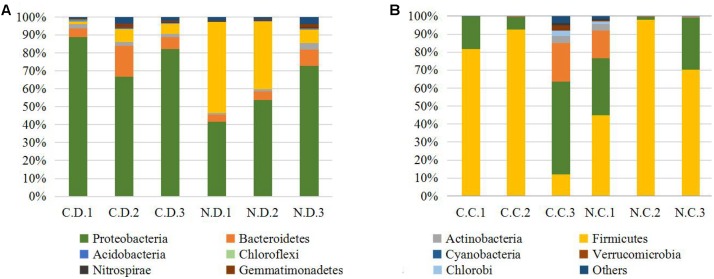
Histogram combination analysis at the bacterial phyla level. **(A)** Duodenum of control chicks (C.D.) and NDV infected chicks (N.D.); **(B)** ceca of control chicks (C.C.) and NDV infected chicks (N.C.).

**FIGURE 2:**
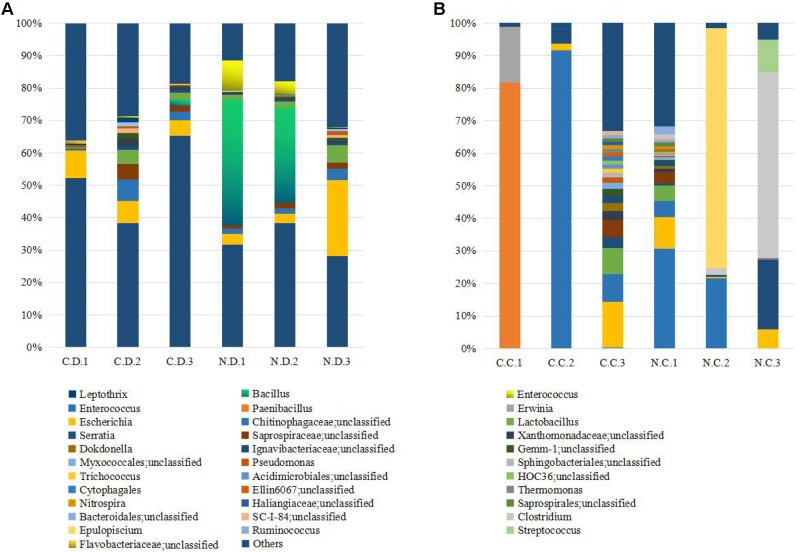
Histogram combination analysis at the bacterial genus level. **(A)** Duodenum of control chicks (C.D.) and NDV infected chicks (N.D.); **(B)** ceca of control chicks (C.C.) and NDV infected chicks (N.C.).

The overall microbial composition for each group at the phylum level was shown in **Figure 1A**. Where bacteria in normal duodenum of 1-day-old chick were identifiable to the phylum level, they belonged predominantly to the Proteobacteria (79.22%), Bacteroidetes (9.59%), Firmicutes (4.16%), and Actinobacteria (2.06%), with Acidobacteria, Chloroflexi, Cyanobacteria, Verrucomicrobia, Nitrospirae, Gemmatimonadetes also identified. When analyzed from the genus level as shown in **Figure 2A**, flora of chick duodenum comprised of *Serratia* (51.97%), *Escherichia* (6.66%), Chitinophagaceae unclassified (3.26%), Saprospiraceae unclassified (2.65%), *Lactobacillus* (2.26%), etc. The duodenum showed relatively homogeneous flora among individuals.

Bacteria in normal ceca of 1-day-old chick were dominated by Firmicutes (87.22%) and Proteobacteria (12.56%), and Bacteroidetes, Actinobacteria, Chlorobi, Gemmatimonadetes, and Nitrospirae were also identified (**Figure 1B**). When analyzed from the genus level, *Enterococcus* (28.78%) *Paenibacillus* (26.91%), *Erwinia* (5.66%), *Escherichia* (5.66%), *Lactobacillus* (2.88%) and *Serratia* (2.76%) accounted for the large part of the cecal flora (**Figure 2B**). The cecal microflora composition varied in different individuals as compared with the duodenal ones.

### Infection of NDV Caused Differential Microbial Diversity in the Duodenal and Cecal Flora

The number of observed OTUs in the duodenum samples of NDV-infected chicks was less than that of the uninfected controls, while those of the ceca samples were almost the same (**Table 1**). NDV infection also showed lower sample richness in duodenal flora as reflected by the abundance index Ace and Chao. Within sample bacterial diversity was assessed by both the Shannon and Simpson analyses, which further demonstrated decreased overall diversity among the duodenal samples from NDV infected chickens as compared with the uninfected controls. The richness and diversity of cecal microflora varied in different individuals as compared with the duodenal ones, and no differences was found between NDV infected and uninfected chicks. Collectively, this data pointed toward a less diverse bacterial population in the duodenal flora from NDV infected chicks.

### The Microbial Composition Was Significantly Altered in NDV Infected Chicks

In order to examine the overall differences in bacterial composition between the NDV infected chicks and the controls, statistical analysis of the community structure at the genus level was performed on the relative abundance of OTUs in each birds. Members of Sinobacteraceae and *Rhodoplanes* were uniformly increased in both the duodenum and ceca samples of NDV infected chicks. On the contrary, NDV infection led to the loss of vast of genera, including members of Xanthomonadaceae, SC-I-84, Cytophagaceae, Bacteroidales, Chitinophagaceae, Gemm-1, Saprospiraceae, Ignavibacteriaceae, Rhizobiales, Sphingobacteriales, Ellin6067, Acidimicrobiales, and *Pseudomonas*, in both the duodenum and ceca samples. Of particular note, the relative abundance of members of Xanthomonadaceae and Cytophagaceae were sharply decreased. Therefore, a uniform of enrichment and depletion of microflora in the duodenum and ceca samples of NDV-infected chicks were characterized.

### NDV Infection Induced Differential Microbial Structures in Duodenum and Ceca of Chicks

Since the microbiota of individuals were significantly enriched or depleted in specific taxa, we examined the differential microbial structures between duodenum and ceca at the genus level after NDV infection in chicks (**Table 2**). The relative abundance of *Serratia* and *Clostridium* were significantly decreased in duodenum, but greatly enhanced in the ceca, suggesting that *Serratia* and *Clostridium* may be translocated from the duodenum to the ceca. On the contrary, we demonstrated a subset of bacteria that possibly translocated from the ceca to the duodenum, including *Dokdonella*, *Lactobacillus*, *Trichococcus, Bacillus, Enterococcus*, and members of Myxococcales, etc.

**Table 2:** Differential bacteria and microbial structure induced by NDV infection in chicks.

Name	Mean (CD)	Mean (ND)	*p*-Value	*q*-Value	Mean (CC)	Mean (NC)	*p*-Value	*q*-Value	
*Sinobacteraceae_unclassified*	0.0037	0.0042	0.353	0.046	0.0027	0.0040	0.008	0.006	↑
*Rhodoplanes*	0.0010	0.0013	0.253	0.034	0.0000	0.0034	0.000	0.000	
*Xanthomonadaceae_unclassified*	0.0096	0.0037	0.000	0.000	0.0099	0.0037	0.000	0.000	
*SC-I-84_unclassified*	0.0062	0.0031	0.000	0.000	0.0037	0.0023	0.004	0.003	
*Cytophagaceae_unclassified*	0.0052	0.0030	0.000	0.000	0.0034	0.0011	0.000	0.000	
*Bacteroidales_unclassified*	0.0046	0.0031	0.005	0.001	0.0037	0.0025	0.009	0.007	
*Chitinophagaceae_unclassified*	0.0324	0.0224	0.000	0.000	0.0305	0.0162	0.000	0.000	
*Gemm-1_unclassified*	0.0089	0.0062	0.000	0.000	0.0076	0.0014	0.000	0.000	↓
*Saprospiraceae_unclassified*	0.0225	0.0158	0.000	0.000	0.0192	0.0112	0.000	0.000	
*Ignavibacteriaceae_unclassified*	0.0072	0.0052	0.001	0.000	0.0078	0.0046	0.000	0.000	
*Rhizobiales_unclassified*	0.0038	0.0029	0.063	0.010	0.0017	0.0008	0.006	0.005	
*Sphingobacteriales_unclassified*	0.0046	0.0035	0.045	0.007	0.0049	0.0015	0.000	0.000	
*Ellin6067_unclassified*	0.0035	0.0029	0.184	0.025	0.0041	0.0024	0.000	0.000	
*Acidimicrobiales_unclassified*	0.0048	0.0040	0.164	0.023	0.0045	0.0004	0.000	0.000	
*Pseudomonas*	0.0060	0.0052	0.172	0.024	0.0071	0.0032	0.000	0.000	
*Serratia*	0.5199	0.3287	0.000	0.000	0.0276	0.0806	0.000	0.000	D↓
*Clostridium*	0.0009	0.0002	0.001	0.000	0.0007	0.2138	0.000	0.000	C↑
*Dokdonella*	0.0035	0.0044	0.076	0.011	0.0085	0.0031	0.000	0.000	
*Lactobacillus*	0.0225	0.0287	0.000	0.000	0.0288	0.0154	0.000	0.000	
*Trichococcus*	0.0022	0.0035	0.004	0.001	0.0046	0.0003	0.000	0.000	C↓
*Myxococcales_unclassified*	0.0031	0.0062	0.000	0.000	0.0068	0.0025	0.000	0.000	D↑
*Bacillus*	0.0065	0.2344	0.000	0.000	0.0032	0.0011	0.000	0.000	
*Enterococcus*	0.0012	0.0469	0.000	0.000	0.2878	0.1687	0.000	0.000	


*C.D., duodenum of control chicks; N.D., duodenum of NDV infected chicks; C.C., ceca of control chicks; N.C., ceca of NDV infected chicks.*

## Discussion

The intestinal immune system in Gallus species must rapidly adapt to colonization by commensal bacteria, as well as the possible entry of pathogenic bacteria. Very low numbers of total bacteria could be counted from the ceca of broiler chickens at hatch as colony-forming units on agar plates (Barnes, 1979; van der Wielen et al., 2002). In contrast, using the cultured-independent 16S rRNA genes Illumina sequencing method, our study showed that much more complex microflora in both the duodenum and the ceca in newly hatched chicks than those in previous studies.

Although it has been generally assumed that the alimentary tract of a chick is devoid of microorganisms, birds examined within only a few hours of hatching frequently contain appreciable numbers of bacteria. It is possible that microorganisms present in the breeder house and incubator are able to transpose the eggshell barrier, reach the albumen, and then become established in the bird intestinal tract during the late stages of embryogenesis (Mead, 1989; Pedroso et al., 2005). Other authors indicate that the acquisition of bacteria occurred immediately after birth from the environment at the hatchery (Lev and Briggs, 1956; van der Wielen et al., 2002). Based on our preliminary analysis of the richness and diversity indexes, the duodenal flora possesses a greater sample richness and higher microbial diversity as compared with that of the ceca flora. Previous studies reported that the bacterial community in the ceca displayed the most diverse in adult chickens (Zhu et al., 2002). Combined with our results, we suggested that the commensal bacteria may enter the digestive tract through the mouth and then implanted in the duodenum at first and enriched in the ceca afterward. The relative homogeneously established microbiota of duodenum among individuals as compared with that of the ceca further supported that the microbiota of duodenum formed earlier. Collectively, the results reported here have confirmed that the bacteria were obtained from the commensal environment immediately at the hatchery.

It has been reported that NDV infection of chickens induced disproportion of GIT microbiome (Li, 2007). This study clearly indicates that NDV infection interferes with the formation of GIT microbiol population in newly hatched chicks. As shown in our study, loss of a subset of bacteria along with decreased richness and diversity were observed in GIT of NDV infected newly hatched chicks. Similar phenomena are also found in many other important poultry diseases like *Eimeria tenella* (Zhou et al., 2017), Marek’s disease virus (Perumbakkam et al., 2014), avian leukosis virus (Ma et al., 2017), etc. The normal microbiota of the GIT of chickens play an important role in inhibiting the establishment of intestinal pathogens (Gomez de Agüero et al., 2016; Günther et al., 2016). Indeed, some important pathogens like *Rhodoplanes* were found to be enriched in both the duodenum and the ceca of the NDV infected chicks. It is resumed that *Rhodoplanes* sp. might be an emerging human pathogen involved in unknown febrile conditions and could cause local infection of any tissues or organs (Zhang et al., 2011). In addition, the duodenum showed relatively homogeneous flora among individuals, but NDV infection made a preference for implantation of known conditioned-pathogens. Similarly, the ceca of normal chicks were dominated by *Paenibacillus* or *Enterococcus* at hatch. Several *Paenibacillus* species produce antimicrobial substances that affect a wide spectrum of micro-organisms such as fungi, soil bacteria, plant pathogenic bacteria, and even important anaerobic pathogens such as *Clostridium botulinum* and *Paenibacillus pasadenensis* (Girardin et al., 2002; Passera et al., 2017). In line with this, more *Epulopiscium* and *Clostridium* established accompanied by the complete loss of *Paenibacillus* after the infection of NDV in newly hatched individuals. *Clostridium* contains around 100 species that include common free-living bacteria, as well as important pathogens. Previous study conferred that bacterial infections could also be enhanced by NDV in a mice model (Hugh et al., 1971), which is consistent with our results. Therefore, these observations as well as previously published studies strongly indicated that NDV infection increased the chance of secondary infection since the intestinal gut might be a great source of conditioned-pathogens.

Bacterial dysbiosis has been linked to altered immune function and/or persistent inflammation. Understanding the roles of microbiota in the intestinal muscosal immunity should offer novel insight into gastrointestinal disease pathophysiology and deliver new immunotherapy strategies (Chang and Lin, 2016). The microbial dysbiosis and translocation is associated with systemic immune activation in HIV and SIV infections, which in turn helps the increase of virus load in the host (Hunt et al., 2011; Marchetti et al., 2011). It is increasingly observed that the NDV infection may be associated with the dysbiosis of gut flora. NDV infection is associated with the mucosal damage in chickens. Thus, it is imperative to better understand the interplay between intestinal microbiota changes and the pathogenesis o of NDV infection.

Overall, the 16S rRNA genes Illumina sequencing technique in this study presented a profile of the early intestinal microbiota in chicks at hatch, and the dysbiosis of bacteria associated with NDV infection. The data will be useful for future studies related to the pathophysiology of NDV in chickens and for experiments evaluating the interactions of NDV and bacteria, and other mixed infections in poultry.

## Ethics Statement

The study protocol and all animal studies were approved by the Shandong Agricultural University Animal Care and Use Committee (SACUC Permission No. AVM140301-19).

## Author Contributions

NC, XH, and ZK collection and assembly of the data, manuscript writing, and data analysis. NC, YH, QH, SY, and LZ discussion, manuscript revision. CX, XZ, and YC concept and design, data analysis, manuscript revision, and final approval of the manuscript.

## Conflict of Interest Statement

The authors declare that the research was conducted in the absence of any commercial or financial relationships that could be construed as a potential conflict of interest.
